# Resolving the Stiffening-Softening Paradox in Cell Mechanics

**DOI:** 10.1371/journal.pone.0040063

**Published:** 2012-07-16

**Authors:** Lars Wolff, Pablo Fernández, Klaus Kroy

**Affiliations:** 1 Institut für Theoretische Physik, Universität Leipzig, Leipzig, Germany; 2 Physik Department, Technische Universität München, Garching, Germany; Dalhousie University, Canada

## Abstract

**Background:**

Despite their notorious diversity, biological cells are mechanically well characterized by only a few robust and universal laws. Intriguingly, the law characterizing the nonlinear response to stretch appears self-contradictory. Various cell types have been reported to both stiffen *and* soften, or “fluidize” upon stretch. Within the classical paradigm of cells as viscoelastic bodies, this constitutes a paradox.

**Principal Findings:**

Our measurements reveal that minimalistic reconstituted cytoskeletal networks (F-actin/HMM) exhibit a similarly peculiar response. A mathematical model of transiently crosslinked polymer networks, the so-called inelastic glassy wormlike chain (iGwlc) model, can simulate the data and resolve the apparent contradiction. It explains the observations in terms of two antagonistic physical mechanisms, the nonlinear viscoelastic resistance of biopolymers to stretch, and the breaking of weak transient bonds between them.

**Conclusions:**

Our results imply that the classical paradigm of cells as viscoelastic bodies has to be replaced by such an inelastic mechanical model.

## Introduction

Cells stiffen upon stretch [1]–[3]. But cells also soften upon stretch [4], [5]. We call this the stiffening-softening paradox of cell mechanics, since both apparently contradictory effects are attributed to the same structural entity or “functional module” [6] of the cell, the cytoskeleton [7]. The cytoskeleton is essentially a semidilute meshwork of semiflexible biopolymers, calling for an explanation by a mechanistic polymer-physics based model [8], [9]. Indeed, *in-vitro* reconstituted cytoskeletal networks were also found to stiffen [10]–[12] *and* soften [12]. Within the classical mechanical paradigm of cells and biopolymer networks as viscoelastic bodies, such contradictory responses constitute a paradox, as they elude attempts of a unified explanation. Accordingly, the different behaviors were previously attributed to distinct network architectures [10]. In the following, we want to challenge this view by revealing that even a passive *in-vitro* cytoskeletal model network exhibits a two-faced mechanical response. Using a simple mathematical model for the inelastic mechanics of a transiently crosslinked biopolymer network, we explain how the apparently paradoxical behavior may naturally emerge from a unified mechanism. Taken together, our results thus show a plausible way of how to resolve the stiffening-softening paradox within a unified framework of inelastic network mechanics, with important implications for cell function, development, and disease [13], [14].

We performed shear rheometry with a biomimetic cytoskeletal model system, an F-actin network isotropically and transiently crosslinked by rigor heavy meromyosin (HMM). The F-actin/HMM system was chosen for its structural simplicity and experimental reproducibility, not for its physiological significance. Its frequency-dependent linear rheology has been well characterized before [15]. Our aim was to demonstrate that even such simple model networks, which are arguably accessible to a schematic mathematical modeling, exhibit a complex two-faced nonlinear rheological response akin to that reported for living cells.

## Results

### Nonlinear Rheology of F-actin/HMM Networks

**Figure 1:**
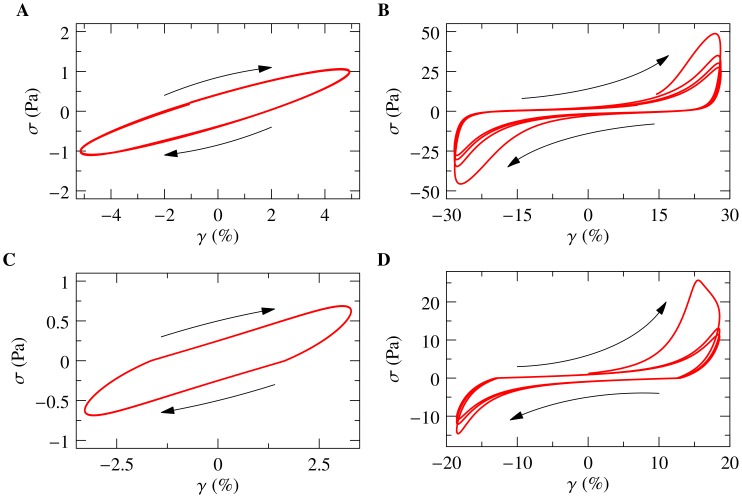
Stress-strain curves for an oscillatory shear strain 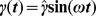. (**a, b**) Experiment: passive transient F-actin/HMM gels (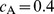 mg/ml, 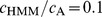) sheared at strain amplitudes of 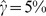 and 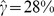, corresponding to a weakly/strongly non-linear response, respectively. The upward bending of the ellipses signals stiffening, their concave regions near maximum strain imply softening. The softening and the ensuing “shakedown” of the stress-strain curves towards a limit cycle are indicative of inelastic fluidization. (**c, d**) Corresponding theory curves from the inelastic glassy wormlike chain (i Gwlc) model [25] (parameters 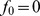, 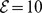, 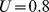, 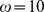 Hz; single-polymer displacement and force were converted to network strain and stress as described in Methods). The absolute stress and strain scales in theory and experiment are compatible on the present (mean-field) level of modeling, but the theory somewhat overestimates the stiffening during the initial large-amplitude loading cycle, and, as a consequence, also the peak force and the strength of the shakedown.

**Figure 2:**
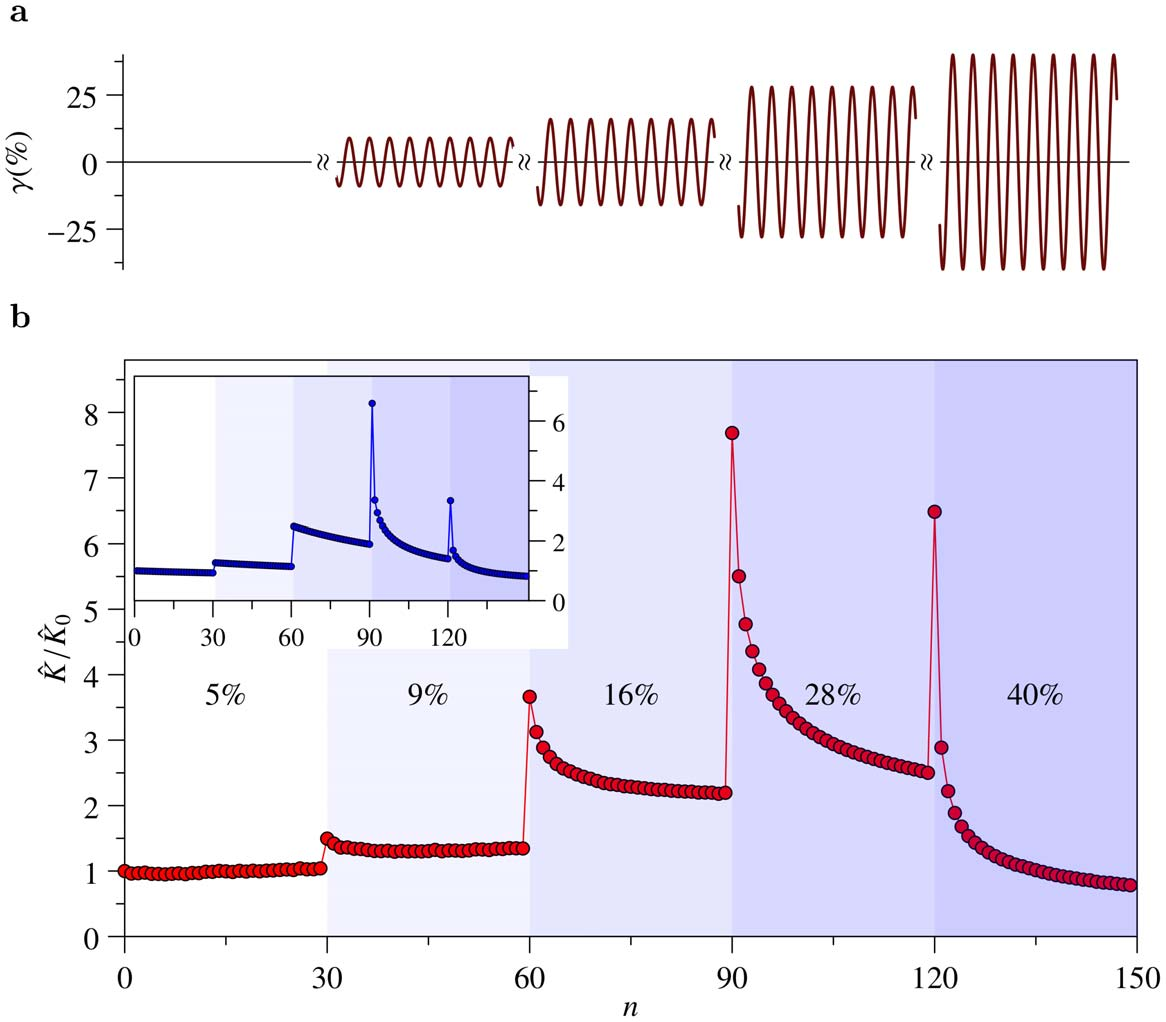
Nonlinear inelastic response of F-actin/HMM networks. (**a**) Schematic of the oscillatory driving protocol (the strain amplitude 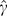 is increased in steps after every 30 cycles, driving frequency 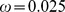 Hz). (**b**) Measured reduced nonlinear modulus 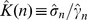 (peak stress over peak strain) as a function of the cycle number 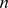. The shaded background indicates the monotonic increase of the strain amplitude 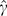 (indicated in percent). Note that the modulus responds nonmonotonically to both transient and stationary loading, hinting at antagonistic mechanisms with multiple time scales. *Inset:* Theory curve from the i Gwlc model [25] reproducing the key features, transient and stationary stiffening and softening with the parameters from Fig. 1 (see also Methods and Fig. E in *Supporting Information S1*).

We applied a staircase of sinusoidal shear excitations. For small amplitude 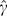, the resulting stress-strain curves have elliptical shapes (Fig. 1a). This means that the stress response 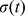 is sinusoidal, like the stimulus 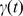, but shifted in phase, as characteristic of a linear viscoelastic (dissipative) response. Upon raising the oscillation amplitude 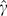 step by step after every 30 cycles (Fig. 2a), deviations from the elliptical shape become increasingly pronounced (Fig. 1b), in line with previous observations for F-actin/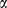-actinin networks [16] and even pure F-actin solutions [17]. Within each cycle, the material stiffens appreciably, which manifests itself in convex stress-strain relations, *i.e.* the ellipses bending upwards. This is the equilibrium viscoelastic stiffening commonly attributed to the nonlinear resistance of individual semiflexible polymers to stretch [9]–[11], [18]. But note that, at the same time, the sample exhibits signatures of softening near the maximum strain 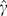, where the stress-strain curves become concave. As a consequence of such repeated softening phases, the maximum stress 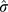 reached in subsequent identical loading cycles decreases continuously until the stress-strain curve settles on a limit cycle. This phenomenon, known as “shakedown” or dynamic softening, is the hallmark of *inelastic behavior*.

To better illustrate how stiffening and dynamic softening interfere, we reduce the full information contained in the nonlinear stress-strain curves in Fig. 1 by introducing a reduced description in terms of the maximum amplitudes 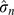 and 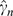 of stress and strain, respectively, for each cycle 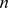. Their ratio 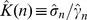 defines a nonlinear modulus as a function of the oscillation frequency and the cycle number 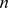, hence of the cycle-to-cycle history of the sample. It captures the essence of stiffening and dynamic softening, while discarding some finer details encoded in the individual stress-strain cycles. The oscillatory staircase protocol with its monotonically increasing amplitude 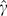 (Fig. 2a) results in a non-monotonic evolution of 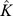 (Fig. 2b). One can distinguish a “transient response” to sudden steps in the driving amplitude 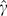
––generically a rapid stiffening followed by a gradual shakedown––from a “stationary response” prospectively attained when the shakedown has ceased after many identical driving cycles. Note that this implies that the modulus 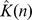 reveals underlying dynamics on multiple time scales. It is a non-monotonic function of the cycle number 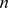 both for the transient and for the stationary response. Such behavior could not easily be explained by a mere elastic stiffening [18]–[20] or softening [21], [22], alone.

**Figure 3:**
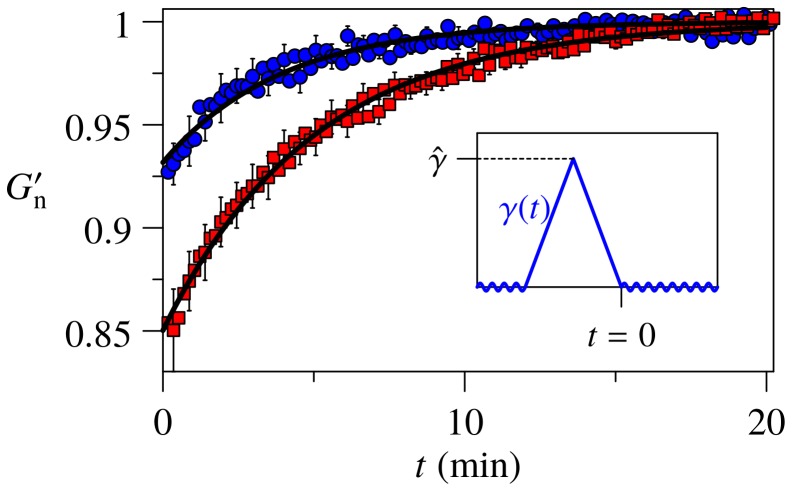
Fluidization and slow mechanical recovery of an F-actin/HMM gel after a transient strain pulse 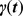 (inset). Stiffness is quantified by the normalized (“n”) real part 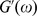 of the linear shear modulus, measured by small sinusoidal oscillations at fixed oscillation frequency 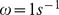, before and after the stretch. The softening immediately after the stretch is found to be sensitive to the maximum strain 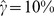 (circles) and 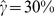 (squares) of the pulse, albeit less pronounced as for cells, where the same pattern is observed at 3–4 times smaller strain amplitudes [4]. Error bars are SE, ensemble size is 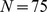; lines represent theoretical (exponential) fits by the i Gwlc model [25]; see Methods and *Supporting Information S1* for further explanations.

It finds a very natural interpretation in terms of an inelastic response, though. To demonstrate this, we adopted a cell rheology protocol aimed at isolating the inelastic contributions to the response by minimizing viscoelastic contributions [4]. The protocol consists of a transient shear pulse of a given amplitude, followed by a recovery phase during which the linear mechanical material properties are monitored over time, as illustrated in the inset of Fig. 3. The main figure depicts the dynamic evolution of the sample stiffness, characterized by the linear storage modulus 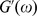, *after* the application of the strain pulse. Right after the pulse, the stiffness of the F-actin/HMM networks is systematically reduced. Similarly to what was previously reported for cells, the effect is sensitive to the amplitude of the pulse (at fixed duration), and the mechanical recovery is slow. The softening is moreover accompanied by an increase in the loss angle (see Fig. F in *Supporting Information S1*). In accordance with the cell-mechanical terminology we thus speak of “fluidization” [4], [23].

### Mathematical Model

The notion of fluidization unifies four of the features described so far: the dynamic softening or shakedown (Figs. 1, 2), the reduction and slow recovery of the modulus after stretch (Fig. 3), and the stationary softening observed in Fig. 2 over long times. For the physical origin of fluidization the transient breaking of weak bonds provides a plausible microscopic mechanism [24]. To support this interpretation, we now turn to a quantitative analysis of our data, based on the inelastic glassy wormlike chain model [25]. The glassy wormlike chain (Gwlc) model is a minimalistic phenomenological model for the Brownian dynamics of biopolymer solutions. It is rooted in the standard polymer-physics model for a semiflexible chain molecule in solution, the wormlike chain (Wlc). But it effectively accounts also for the caging and enthalpic trapping of such a polymer by the surrounding polymer matrix, resulting in a microscopic mechanical susceptibility 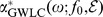, depending on the frequency 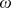, prestressing force 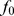, and a stretching parameter 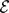, interpreted as a characteristic bond breaking enthalpy in units of the thermal energy 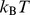. The Wlc and the Gwlc can parametrize a wealth of mechanical data obtained in single molecule experiments [26] and rheometric measurements of biopolymer solutions, networks and cells [27]. The *inelastic* Gwlc (i Gwlc) adds to this an effective description of bond kinetics [25], i.e. it is applicable to nonequilibrium situations characterized by an appreciable dynamical evolution of the bond network mutually connecting the polymers (see Fig. D in *Supporting Information S1* for an illustrative sketch). This is realized by introducing a dependence of the microscopic susceptibility 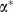 on the mean fraction 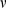 of closed bonds, 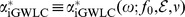. To keep the model as simple as possible, we limit our discussions to “inelastic” (as opposed to “plastic”) deformations by requiring reversible binding-unbinding kinetics. Broken bonds ultimately reform in their original equilibrium states after the external load has been released. This means that we refrain, at the present stage, from distinguishing between the breaking of sacrificial bonds that triggers a transient domain unfolding [28] and the breaking and reforming of cytoskeletal filaments [29] or the unbinding and rebinding of their mutual sticky contacts [30], crosslinkinking molecules [15], [16], or actin-myosin cross bridges [31].

A more detailed description of the model is given in *Supporting Information S1*. For the sake of our present discussion, its essential predictions for the shear modulus 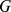 are (*i*) a roughly linear increase with prestress if bond-breaking is negligible, 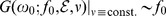, (*ii*) a reciprocal relation to the number of bonds at constant stress, 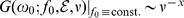, with 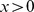, and (*iii*) bond softening under stress. The latter is implemented by a Bell-type exponential force dependence of the bond opening and closing rates, 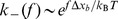, 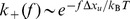 with the widths 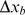 and 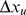 of the bound and unbound state.

## Discussion

The interplay between the nonlinear mechanical response of individual polymers and the slow but stress-sensitive bond dynamics gives rise to a rich and complex mechanical behavior of the i Gwlc. It naturally predicts the bent stress-strain curves and their softening characteristics and gradual shakedown, as exemplified in Fig. 1d, and the fluidization and slow recovery after a transient strain pulse (Fig. 3, lines). Even the stiffness evolution on multiple time scales, depicted in Fig. 2, is well reproduced by the model (inset). Here, we always considered the prestressing force 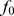 as a (small) constant. It represents frozen-in stresses in the network, which are supposedly weak for our passive reversibly crosslinked networks. But we note in passing that 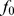 might play a much more dynamic role in applications of the i Gwlc to cell rheological data, where it might under certain circumstances be needed to represent an active contractile cell response.

Beyond providing an economical parametrization of our own data and known literature results [4], [5], [10]–[12], [30], [32], the i Gwlc makes a plausible and intuitive quantitative proposal for the underlying molecular mechanism. More precisely, by analyzing the model equations, stiffening can be attributed directly to the characteristic nonlinear stretch response of individual semiflexible biopolymers, causing a prompt viscoelastic response to an applied stress. Softening emerges as an aftermath to an applied strain from the slow and stress-sensitive dynamical evolution of the mutual bonds between the biopolymers, and is therefore better characterized as an inelastic fluidization. The time-scale separation between viscoelastic stiffening and bond softening turns out to be at the heart of the observed complex nonlinear dynamical response, because large internal stresses can build up before eventually relaxing via inelastic bond breaking. The stationary effects, in contrast, rely on a static balance between polymeric stiffening and bond breaking. The model quantitatively relates these essential properties to each other and also to other characteristic features of the mechanical response of biopolymer networks and cells. For example, scale-free power-law spectra, as observed in cell rheology [33], are a characteristic feature of the model (see Ref. [27] and *Supporting Information S1*). Finally, the i Gwlc makes a number of interesting testable predictions for future investigations. For instance, as a direct consequence of the Bell-type stress-dependence of the bond strength, we find that the peak force reached in a large strain ramp or pulse grows essentially logarithmically with the characteristic rate at which the force increases. Conversely, the fraction of broken bonds––and therefore the resulting fluidization of the sample––is quite insensitive to the duration of the stimulating pulse, over a broad range of time scales. This particular feature has indeed already been demonstrated for live cells [5]. However, beyond a certain effective “yield threshold”, the bond fraction sensitively depends on the (imposed or attained) maximum strain, no matter what the yield force is (see *Supporting Information S1* for a quantitative description).

**Figure 4:**
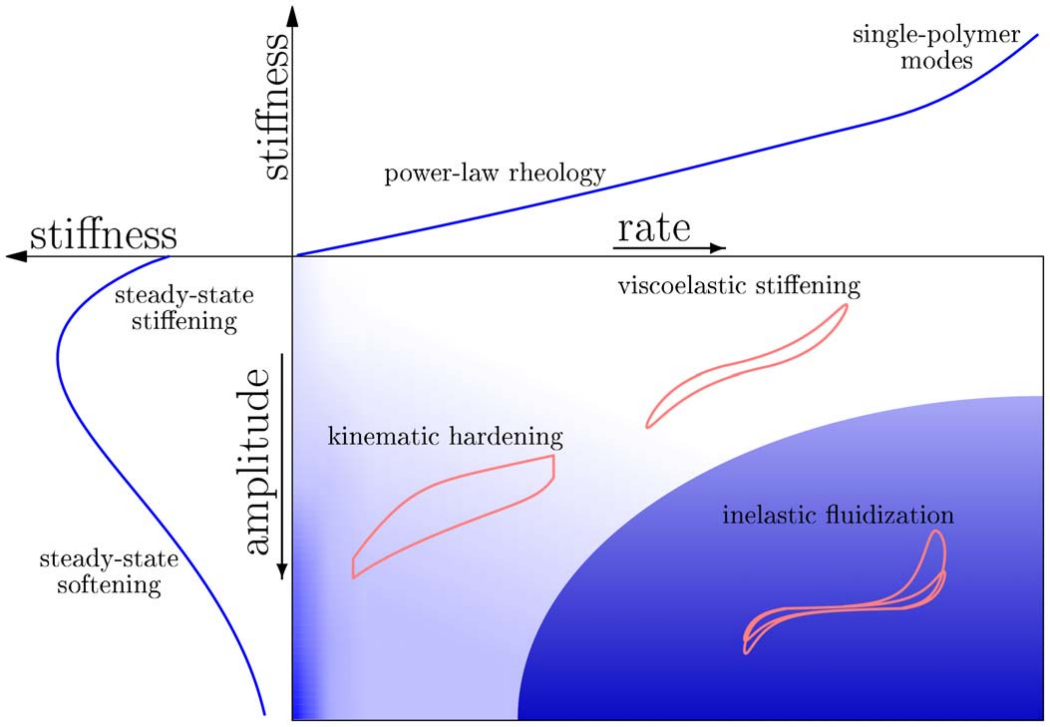
Constitutive diagram for the iGWLC model. The central panel gives a qualitative graphical summary of the mechanical response predicted by the model as a function of the amplitude and characteristic rate of an imposed deformation pulse. At low amplitudes, in the linear regime, it exhibits power-law rheology (upper panel, log-log scale). At low rates, in the quasistatic regime, it exhibits stiffening at low amplitudes, where entropic stiffening of the polymer backbone dominates, and softening at high amplitudes, where the stiffening is eventually overruled by the exponential bond softening (left panel, linear scale). This mechanism underlies the initially ascending and later descending steps in the nonlinear modulus in Fig. 2. At high rates *and* high amplitudes, a steep initial stiffening with subsequent fluidization and slow recovery governs the response (central panel). The schematic stress-strain curves for oscillatory driving exemplify the salient features of the nonlinear response in the various parameter regions.

The observation that the rate and amplitude of an imposed deformation affect the nonlinear response so differently suggests to delineate a non-equilibrium *constitutive diagram* in the reduced parameter plane spanned by the rate and amplitude of an imposed deformation (Fig. 4 central panel). The background shading and the small representative stress-strain cycles distinguish domains of deformation rate and amplitude with a qualitatively distinct mechanical response. The limiting behaviors at vanishing rate and vanishing amplitude, i.e. near to the coordinate axes, are further characterized in the side panels (note the different labelings on their outer axes). The upper panel depicts the rate-dependent viscoelastic response for vanishing amplitude, hence essentially the linear frequency-dependent shear modulus on a log-log scale, exhibiting power-law rheology. The left panel (linear axes) shows the nonlinear shear modulus in the limit of slow driving. Note the turnabout from inelastic stiffening to softening in response to a quasi-static driving, which is responsible for the initially ascending and later descending plateaus in the nonlinear modulus 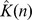 in Fig. 2. This non-monotonic stationary stress-stiffness relation originates in the sigmoidal sensitivity of the bond fraction to the force (see *Supporting Information S1*). For slightly larger rates, the stiffening becomes steeper, which gives rise to a “kinematic-hardening” type behavior (central panel). Finally, if the deformation rate and amplitude of the loading are both large, the response features steep initial stress stiffening and ensuing dynamic fluidization, as caused by the amplitude steps in Fig. 2, as well as the fluidization-recovery pattern illustrated in Fig. 3. The depicted representative stress-strain cycle exhibits shakedown, as in Fig. 1. Though it should not be confused with a thermodynamic state diagram, the constitutive diagram in the central panel of Fig. 4, if judiciously interpreted, can serve as a compact characterization of the multifaceted nonlinear mechanical response of transiently crosslinked biopolymer networks and as a potentially useful road map for cell rheologists.

In summary, we have explored the stiffening-softening paradox of cell mechanics, both by rheological measurements of minimal cytoskeletal model systems (F-actin/HMM) and by theory. The nonlinear nonequilibrium mechanical response of the reconstituted networks was found to provide a close match to previous cell rheological measurements, albeit at 3–4 times larger amplitude. It was moreover well parametrized by the inelastic glassy wormlike chain model, which suggests a unified mechanistic explanation. Stiffening, as a direct *viscoelastic response* to an applied stress, is attributed to the characteristic nonlinear stretch resistance of individual semiflexible biopolymers. Conversely, softening emerges as an aftermath to an applied strain from the dynamical evolution of the mutual bonds between the biopolymers, and it is better characterized as an *inelastic fluidization*. By emphasizing the key role played by inelastic processes, the proposed polymer-physics based explanation of the stiffening-softening paradox clearly transcends the classical mechanical paradigm of biopolymer networks and cells as viscoelastic bodies. Our unifying explanation based upon inelastic processes is specific concerning the basic mechanism, yet robust against details of its implementation. It makes reference to microscopic elements in the molecular structure of the cytoskeleton, such as biopolymers and their mutual transient bonds, and relates them quantitatively to a wide range of rheological responses. It would also be straightforward to accommodate more sophisticated physical constituents accounting for dynamic prestresses generated by molecular motors, stress-induced domain unfolding, or catch bonds. In the minimalistic implementation discussed here, our model is, at the same time, still schematic and deliberately employs bold simplifications. In particular, it does not address network and crosslinking geometries, nor is it parametrically fine-tuned to a particular molecular architecture, as would be required for extracting reliable parameter values (such as binding affinities of crosslinkers) from fits to experimental data. In return, one may hope that it can qualitatively capture major elements of the mechanical phenomenology of both networks *and* cells, irrespective of their utterly different degrees of molecular complexity.

## Methods

### Protein Preparation and Rheology

Passive rigor F-actin/HMM networks at various concentrations were prepared as previously described [34], except that no gelsolin was added. Nonlinear oscillatory experiments were performed at actin concentration 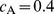 mg/ml and HMM molar ratio 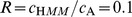. Data for pulsed loading were pooled over two different actin concentrations 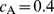 mg/ml and 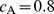 mg/ml and values of 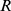, 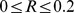, see Table A in *Supporting Information S1* for details. We used a commercial AR G2 shear rheometer (TA Instruments, New Castle, USA) in cone-plate geometry (40 mm diameter, cone opening angle 1°). About 370 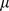
l sample were loaded within 1 minute into the rheometer. The transition to rigor HMM upon ATP depletion is followed by recording the elastic response of the F-actin/HMM network over time (see Fig. B in *Supporting Information S1*). Two different rheological protocols were applied. The first protocol, termed “nonlinear oscillations”, consisted of shear oscillations at 0.025 Hz with a staircase increase in the amplitude. The amplitude was kept constant for 30 cycles and then increased to a higher value, where it again was kept constant for 30 cycles, and so forth. Amplitude values were 5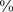, 9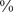, 16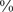, 28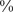, and 40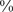 (cf. Fig. 2a). The second protocol, termed “fluidization protocol”, was chosen to specifically probe the inelastic contribution to the response, as pioneered in cell rheology [4]. A triangular shear pulse of four minutes duration and variable amplitude was applied to the sample, followed by a waiting time of at least 20 minutes. During the waiting time, the linear frequency-dependent modulus was constantly recorded by applying small shear oscillations of 1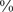 amplitude at frequencies of 2 Hz (cf. Fig. 3).

### Data Analysis

For the nonlinear oscillations, raw data were extracted from the rheometer. Spline smoothing was applied to the data using a custom-made Python script. Torque 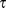 and angle 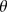 were converted to stress 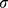 and strain 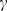 using 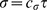 and 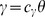, with conversion factors of 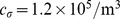 and 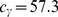, respectively, which are calculated from the cone geometry. From the resulting stress-strain data, a nonlinear modulus 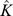 was calculated as described in the main text. The response to the nonlinear oscillations was qualitatively reproducible among different samples (Fig. E in *Supporting Information S1*). No averaging over samples was performed.

To relate theoretically calculated filament forces 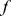 to network strains 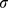, we used the relation [35] 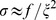, with the mesh size [36] 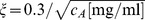; an estimate for the strain was obtained by normalizing the displacement by the mesh size 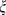.

For the pulsed loading experiments, the linear stiffness responded to a strain pulse by a systematic decrease followed by a recovery. Often, the recovery did not reach the pre-shear value. The failure to fully recover is probably due to slow, uncontrolled network reorganization processes, because, independent of the shear pulses, the modulus always exhibited a slow, nearly linear drift (Fig. B in *Supporting Information S1*). To correct for this “background drift”, the data were parametrized by a linear function 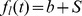 at late times 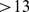 min, where all curves were to a good approximation linear in time. The data were then normalized by this linear asymptote (Fig. C in *Supporting Information S1*), 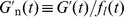 and then averaged over 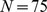 samples.

Statistical analysis of the recovery data was performed using a Monte-Carlo resampling bootstrapping method, as described in the following. For each time step, the respective values of 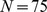 normalized experimental curves were pooled. A resampled curve was created by drawing (with replacement) one value from this pool for every time step. To a total of 1000 resampled recovery curves, exponential functions.
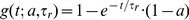
(1)were fitted, with the value 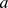 of the normalized stiffness after stimulus cessation and recovery time 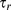. We obtained 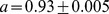 and 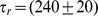
s for 10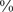 pulse amplitude and 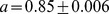 and 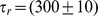
s for 30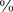 pulse amplitude. Errors are standard errors of the mean. Note that the recovery time for the large pulse is larger than the respective time for the small pulse. This behavior is actually expected from theoretical considerations (*Supporting Information S1*).

### Model

The inelastic glassy wormlike chain (i Gwlc) [25] is an extension of the (equilibrium) glassy wormlike chain (Gwlc) model, which, in turn, is a phenomenological extension of the wormlike chain (Wlc), the standard coarse-grained mathematical description of an individual semiflexible polymer in solution [37]. Beyond the common Wlc, the equilibrium Gwlc phenomenologically accounts for the caging and trapping of a test polymer by the surrounding polymer network. The corresponding slowdown of the long wavelength bending undulations of the polymer backbone is, in mathematical terms, represented by a stretching of the ordinary Wlc relaxation spectrum. Beyond a characteristic minimum interaction wavelength 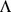 (on the order of the entanglement length) the relaxation times 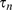 of all Wlc modes 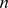 of wavelength 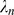 are modified by a mode-dependent Arrhenius factor
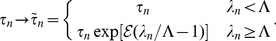
(2)


This modification of the relaxation spectrum gives rise to a dramatic slowdown of the dynamics at long times or small frequencies [37], producing power-law rheology with a small apparent power-law exponent 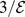 [27], as ubiquitously observed for cells [7].

A pertinent example for an observable characterizing the mechanical response under an optional prestressing force 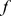 is the complex microscopic susceptibility to transverse displacements [37], given by
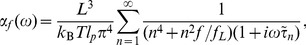
(3)with the polymer length 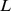, persistence length 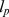, Euler buckling force 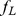, and thermal energy 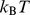. In the limit of infinitely long polymers, the expression becomes independent of the length and can be converted to an integral. Note that equation (3) implicitly depends on 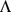 and therefore is better written as 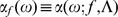. To evaluate the force response to a given strain stimulus 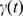 in linear response, a superposition principle can be used,
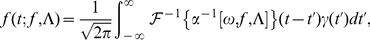
(4)where 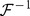 denotes the inverse Fourier transform.

In the *inelastic* Gwlc (i Gwlc) model, we interpret 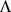 as the average backbone length between adjacent bonds of the test chain with the background network, and 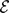 as the height of the free energy barrier (in units of thermal energy) that has to be overcome to break a bond. In contrast to the equilibrium Gwlc, 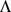 is not assumed to be a fixed equilibrium quantity 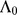, but is allowed to evolve with time (Fig. D in *Supporting Information S1*). It is related to the state variable 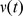, describing the mean fraction of closed bonds (or “bond fraction”) at a given time 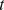, by 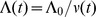. The equilibrium Gwlc is recovered as the special case of a fixed average bond fraction 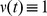.

The bond fraction evolves according to a simple generic first-order kinetic equation,
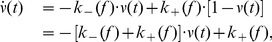
(5)where 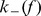 and 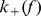 are force-dependent off- and on rates, respectively. The transition rates are taken to depend exponentially on the polymer backbone tension 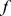 in the standard way [38],
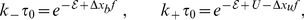
(6)where 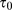 is a characteristic time scale that depends on the properties of the binding potential, 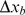 and 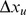 are the widths of potential wells corresponding to the bound and unbound state, respectively, and 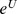 is the relative binding affinity [38], [39].

To summarize, the model combines two fundamental nonlinear mechanical paradigms, namely single-polymer stiffening and a transient bond softening under load. The resulting nonlinear response is evaluated numerically using a nonlinear update scheme implemented in C++. In brief, in each time step 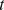, the bond fraction 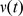 is updated according to equations (5) and (6) and the force history 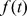. Both 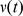 and 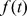 then determine the Gwlc response, at a given time 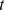, via the Gwlc mode spectrum 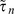 [25].

## Supporting Information

Supporting Information S1 The Supporting Information S1 covers some additional technical details concerning the data analysis and the theoretical model, and auxiliary numerical and experimental data.
(PDF)
