# Supplementary material for: Resolving the Stiffening-Softening Paradox in Cell Mechanics
Source: PLoS One. 2012 Jul 16;7(7):e40063. doi: 10.1371/journal.pone.0040063 (PMC3397976; doi:10.1371/journal.pone.0040063)
Supplement: Supporting Information S1 — The Supporting Information S1 covers some additional technical details concerning the data analysis and the theoretical model, and auxiliary numerical and experimental data. (PDF) [file pone.0040063.s001.pdf]

Resolving the stiffening-softening paradox in cell mechanics

Lars Wolff[1], Pablo Fernandez[2], and Klaus Kroy[1]

S-1 Rate-dependent yield force, amplitude-dependent fluidization

In a previous article [1], it was shown that the inelastic GWLC can account for fluidization. In the model, fluidization is concomitant with a particular shape of the stress-strain curve, which resembles a plastic yielding event. It is interesting to note that in the F-actin/HMM experiment, stiffening is much weaker than predicted by the theory (see figure 1 in the main text), which may arguably be attributed to additional slip in the network. This deviation is only a *quantitative* one which leads to a more diffuse, less sharp yield threshold and under certain conditions to a less drastic fluidization. Still, none of the qualitative effects discussed below depends on a particularly strong stiffening.

For clarity, we briefly summarize the inelastic yielding mechanism. In the stress-strain curves, the characteristic signature is a softening (see also figures 1c and d in the main text). The reason of this softening is a force-induced bond breaking ("yielding") at $f = f_y$. The *yield force* f_y is not a prescribed parameter of the model, but an emergent property determined by a dynamic balance of single-polymer stiffening and bond softening. It is well-defined in the limit of a strong initial time-scale separation between the mechanisms of softening, stiffening, and the experimental protocol. At low to intermediate driving rates, the delay of the viscoelastic stiffening with respect to the driving is negligible, so that we only have to consider the relation between the time scale T_{pulse} set by the experimental protocol and and the average time k_-^{-1} for thermal bond opening. A yielding behavior is exhibited whenever the initial bond time scale $k_-(t = 0)^{-1}$ is much smaller than the experimental time scale T_{pulse}, $k_-(t = 0) \cdot T_{\text{pulse}} \ll 1$. In this case, the bond fraction initially hardly responds to the stimulus. For large enough amplitudes, the viscoelastic stiffening therefore prevails, leading to high forces. Increasing the driving amplitude even more, the exponential force dependence of the transition rates (see SOM text below) will strongly reduce the current bond opening

[1]Institute for Theoretical Physics, University of Leipzig, Postfach 100920, 04009 Leipzig, Germany
[2]Department of Physics, TU München, James-Franck-Str., 85748 Garching, Germany

time scale $k_-(f)^{-1}$, leading to sudden yielding events. This provides an implicit operational definition of the yield force f_y by

$$k_-(f_\mathrm{y})^{-1} \approx f_\mathrm{y}/\dot{f}. \tag{1}$$

Here, we estimated the experimental time scale as the yield force f_y divided by the average force rate $\dot{f}$. Solving this for f_y gives [1]

$$f_\mathrm{y}\Delta x_b \approx \mathrm{W}\left(\mathrm{const.} \times \Delta x_b \tau_0 e^{\mathcal{E}} \dot{f}\right), \tag{2}$$

which reveals the (essentially) logarithmic *rate dependence* of the yield force (SI Fig. Aa), since the Lambert-W function $\mathrm{W}(x)$ is well approximated by a logarithmic function at large x. From the above discussion, it is also apparent that the yield force must be *amplitude independent* (Fig. Ab). At the smallest force fulfilling condition (1), the material yields, no matter what the stimulus amplitude is. Given this amplitude independence, we can use the yield force f_y to operationally distinguish "small" from "large" stimuli. "Large" are those stimuli that cause the force f to reach the yield threshold f_y.
Interestingly, the opposite behavior is found for the fluidization caused by the stimulus. We quantify the degree of fluidization by the minimal value ν_min of the bond fraction reached during the pulse. It hardly depends on the force rate over a broad range of rates, but is strongly influenced by the stimulus amplitude (Fig. Ac & d), as also observed in experiments [3, 4]. The amplitude dependence is easily explained by noting that increasing the amplitude at fixed rate implies increasing the pulse duration accordingly. This in turn means that the bonds are longer subjected to the external force and thus have more time to open. Note that this argument breaks down if the pulse duration is so long that the bond fraction can equilibrate under the external force within the duration of the experiment. As a consequence, the amplitude dependence saturates. The saturation value can be estimated as the stationary bond fraction $\nu_\mathrm{st}(f)$ under the yield force f_y, $\nu_\mathrm{st}(f_\mathrm{y}) = [1 + \exp(-U + f/f_T)]^{-1}$. For the examples given in Fig. Ad, this value is nearly zero, due to the large yield force. Much less intuitive is the very weak rate dependence of the bond breaking. Yet, it can be rationalized by the following order-of-magnitude estimate. To establish relation (1), we noted that for yielding to take place, the inverse bond off rate $k_-(f_\mathrm{y})^{-1}$ at the yield force has to be on the order of the pulse duration $T_\mathrm{pulse} \sim k_-(f_\mathrm{y})^{-1}$. Given the amplitude independence of f_y, we can roughly estimate the change $\Delta\nu$ in bond fraction as $\Delta\nu \sim T_\mathrm{pulse} \cdot k_-(f_\mathrm{yield}) \sim T_\mathrm{pulse}/T_\mathrm{pulse} = \mathrm{const.}$ While the argument requires small $\Delta\nu$, it apparently captures the underlying physics well beyond that limit, as demonstrated in Fig. Ac.

S-2 Recovery of the inelastic GWLC model after a transient strain pulse

In the main text, we found that the time scale of recovery of the linear stiffness following a transient strain depends on the amplitude of the strain pulse (Fig. 3). This behavior can be

understood from theoretical considerations, as shown below.
For slow bonds, the single-polymer relaxation time τ_{L_e} ($\tau_{L_e} \approx 0.12$s for $l_e = 1.6\mu$m, $l_p = 10\mu$m, and solvent friction per length $\zeta = 0.07$pN/μm^2) is much smaller than the zero-force bond recovery time $\tau \equiv (k_+ + k_-)^{-1}$. The force is then almost constant during most of the relevant time *after* stimulus cessation. The bond fraction will therefore relax nearly exponentially. A second consequence of the approximately constant force in the time period of interest is that the stiffness G' only changes due to the recovery in ν. G' is a monotonically increasing function of ν [1]. The dependence of G' on ν can thus be approximated by a power law over not too large ranges of ν,

$$\Delta G' \sim (\Delta\nu)^{x_{\text{eff}}}, \tag{3}$$

replacing the actual ν-dependence by the best-fitting power law over the range considered. As the approximation is only defined locally, the actual value of x_{eff} depends on the particular range of ν. Given the exponential recovery of ν with time constant τ,

$$\Delta\nu \sim e^{-t/\tau}, \tag{4}$$

the stiffness also recovers nearly exponentially as

$$\Delta G'(t) \sim (\Delta\nu)^{x_{\text{eff}}} \sim e^{-t/(\tau/x_{\text{eff}})} = e^{-t/\tau_{\text{eff}}}, \tag{5}$$

with the effective time constant $\tau_{\text{eff}} \equiv \tau/x_{\text{eff}}$.
Because we consider the recovery after pulsed loading, the range of ν is bounded from above by the force-free steady-state bond fraction ν_{st}, which is independent of the pulse amplitude. From below, ν is bounded by the amplitude-dependent value $\nu(t = 0)$ immediately after the pulse, $\nu(t = 0) \leq \nu(t) \leq \nu_{\text{st}}$. Because of the amplitude-dependent ν range, the effective exponent also depends on the amplitude, as discussed above. The dependence of G' on ν gets steeper [1] for higher ν, implying that the effective exponent x^{10}_{eff} for 10% strain amplitude is larger than the effective exponent x^{30}_{eff} for 30% strain amplitude, $x^{10}_{\text{eff}} > x^{30}_{\text{eff}}$. From this relation, we obtain the following relation for the effective relaxation times

$$\tau^{10}_{\text{eff}} = \frac{\tau}{x^{10}_{\text{eff}}} < \frac{\tau}{x^{30}_{\text{eff}}} = \tau^{30}_{\text{eff}}. \tag{6}$$

We thus expect the recovery time measured for 30% pulse amplitude to be *larger* than for 10% pulse amplitude, consistent with our experimental data (see main text and SOM text above).

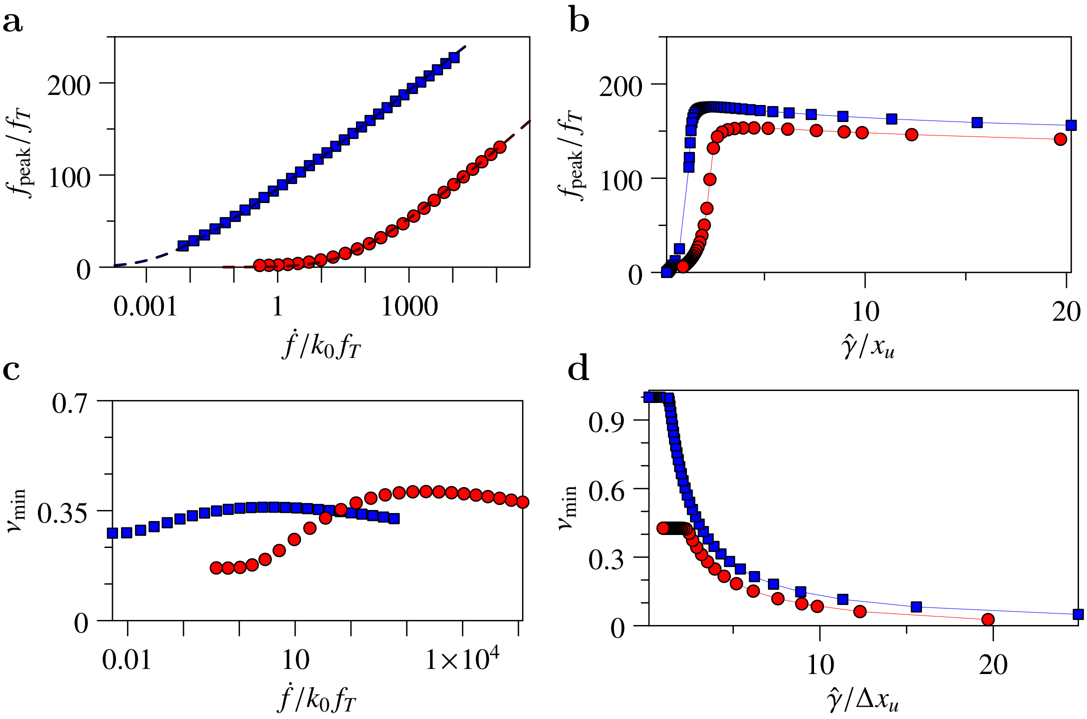

Figure A. Characteristic yielding predicted by the inelastic GWLC model. Rate and amplitude (in-)dependences of peak force $f_{\rm peak}$ and peak fluidization $\nu_{\rm min}$ reached during a deformation pulse. The peak force reached during a large deformation pulse can be interpreted as the yield force f_y. (**a**) Nearly logarithmic dependence of the peak force on the average force rate (symbols). Dashed lines are fits by equation (2) with prefactors 1.5 and 1.4 and scaling constants 1.4 and 0.4 for squares and circles, respectively. (**b**) Once the yield force is reached, the peak force is nearly independent of the amplitude. Lines are guides to the eye. (**c**) Weak dependence of the minimum bond fraction during the pulse on the average force rate. (**d**) Minimum bond fraction during the pulse *vs* pulse amplitude. Lines are guides to the eye. For **a** and **c**, rates are given in terms of $k_0 = k_+(f=0) + k_-(f=0)$, peak force is given in terms of $f_T = k_B T/(\Delta x_u + \Delta x_b)$, and amplitudes are $\hat{\gamma}/\Delta x_u = 4.1$ (squares) and $\hat{\gamma}/\Delta x_u = 3.3$ (circles). For **b** and **d**, pulse durations are $T_{\rm pulse} k_0 = 2$ (squares) and $T_{\rm pulse} k_0 = 0.27$ (circles). Parameters are chosen to represent a stiff (squares) and a soft (circles) material, respectively. The parameter sets differ in the following values: $\mathcal{E} = 14.6$, $U = 8.6$, $f_0 = 0.48$ (stiff) and $\mathcal{E} = 12.2$, $U = -0.3$, $f_0 = 0.16$ (soft); f_0 represents an internal prestressing of the polymers that does not affect the bond kinetics. Global parameters: $l_p = 10$ and $\zeta_\perp = 0.07$, consistent with common literature values for F-actin [2] if length is measured in μm and time is measured in s. We further fix $\Lambda_0 = 1.62$, which is consistent with this convention if we identify Λ_0 with the entanglement length. Δx_u is self-consistently defined as the value of the standard deviation of the equilibrium fluctuations of the contour at the entanglement time $\tau_{L_e} = \zeta_\perp L_e^4/k_{\rm B} T l_p \pi^4$, $\Delta x_u = \sqrt{{\rm MSD}(\tau_{L_e}; \mathcal{E}, f_0)}$ ($\Delta x_u = 0.053$ and 0.067 for stiff and soft, respectively). The width Δx_b of the bound state was arbitrarily set to a small value of $\Delta x_b = 0.005$.

R	c_A/(mg/ml)	# 10%	# 20%	# 30%	$\sum$
0	0.4	2	0	0	2
0.01	0.4	1	2	2	5
0.02	0.4	1	2	2	5
0.03	0.4	2	2	2	6
0.04	0.4	2	2	2	6
0.05	0.4	12	12	12	36
0.05	0.8	1	2	2	5
0.1	0.4	2	2	0	4
0.2	0.4	2	2	2	6
$\sum$		25	26	24	75

Table A. Number of datasets (#) used for the pulsed loading experiments for 10%, 20%, and 30% strain amplitude for various values of the actin concentration c_{A} and molar ratio $R = c_{\mathrm{HMM}}/c_{\mathrm{A}}$.

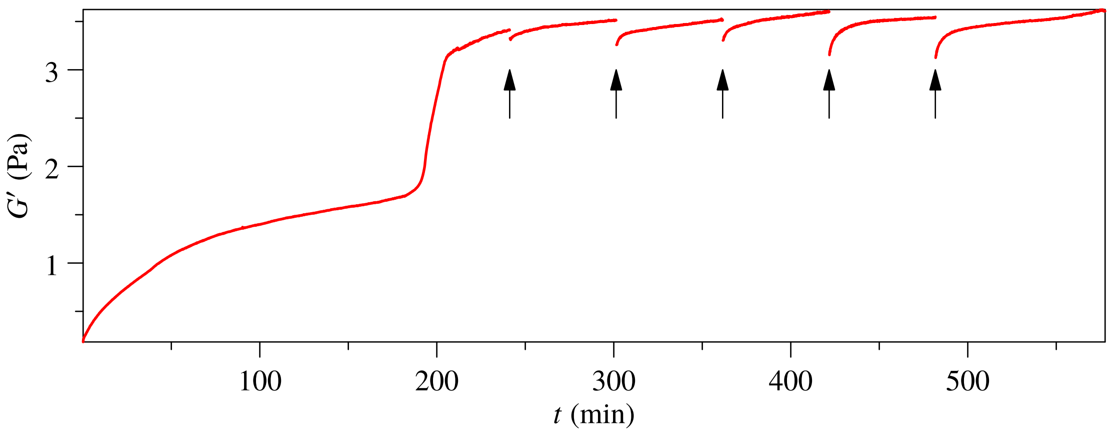

Figure B. Exemplary pulsed loading experiment. At zero time, the sample is loaded into the rheometer. The subsequent increase in stiffness is a symptom of actin polymerization. The shoulder at $t = 200$ min indicates ATP depletion, where HMM enters its rigor state. Arrows mark times of shear pulse application (10 %, 20 %, 20 %, 30 %, 30 % amplitude, from left to right). The linear storage modulus G' is evaluated at 2 Hz. The decrease of the modulus triggered by the pulse and the subsequent recovery is superposed by a slow linear increase. $c_{\mathrm{A}} = 0.4$ mg/ml, $c_{\mathrm{HMM}}/c_{\mathrm{A}} = 0.02$.

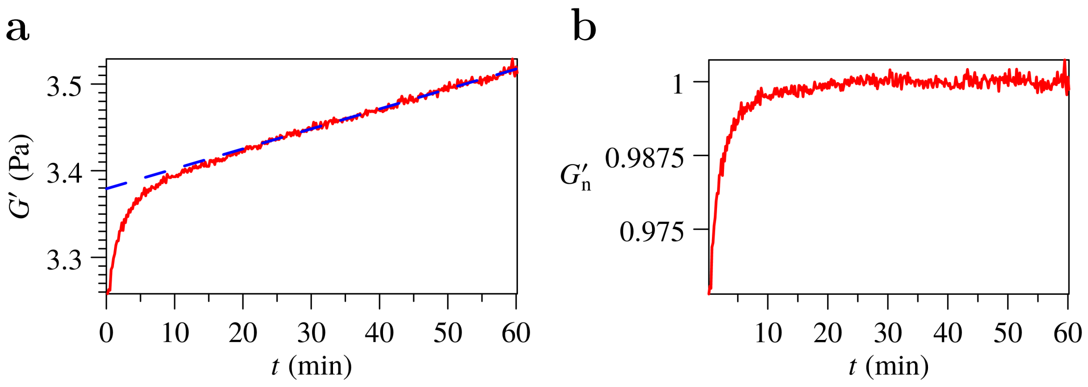

Figure C. Recovery of the linear modulus after a transient shear pulse. Zero time marks cessation of the strain pulse. (**a**) Unnormalized data (solid red line) together with a linear fit to the slope during the last 47 min (blue dashed line). (**b**) Division of the unnormalized data by the fitted linear function yields the normalized data. Conditions were $c_A = 0.4$ mg/ml, $c_{\mathrm{HMM}}/c_A = 0.02$, and $\hat{\gamma} = 20\%$.

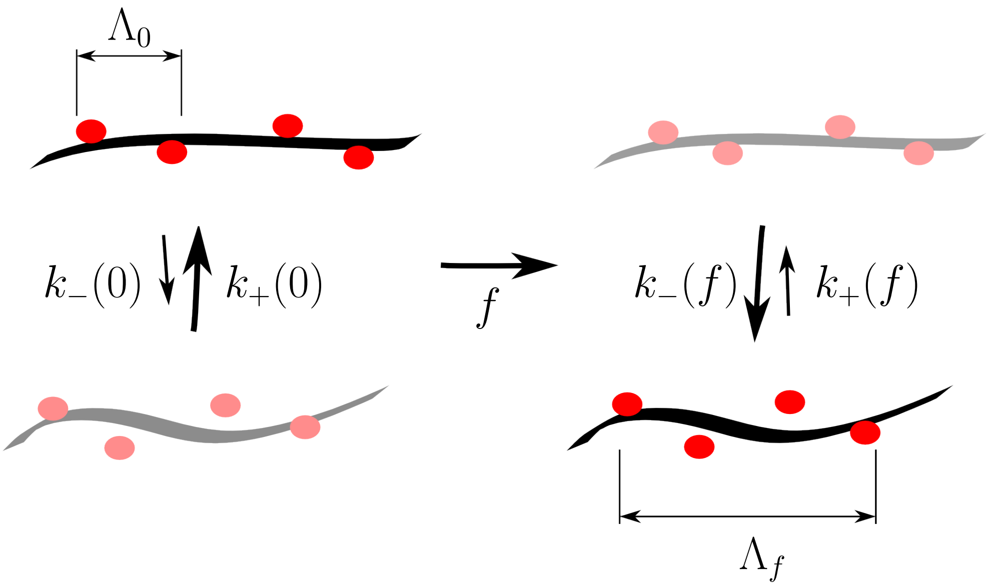

Figure D. Reversible bond breaking. The polymer is decorated by transient bonds to the background network. Bonds open and close with force-dependent rates k_- and k_+, respectively, giving rise to an average distance Λ between closed bonds. Applying a force f disturbs the equilibrium between binding and unbinding, and thus changes the value of Λ.

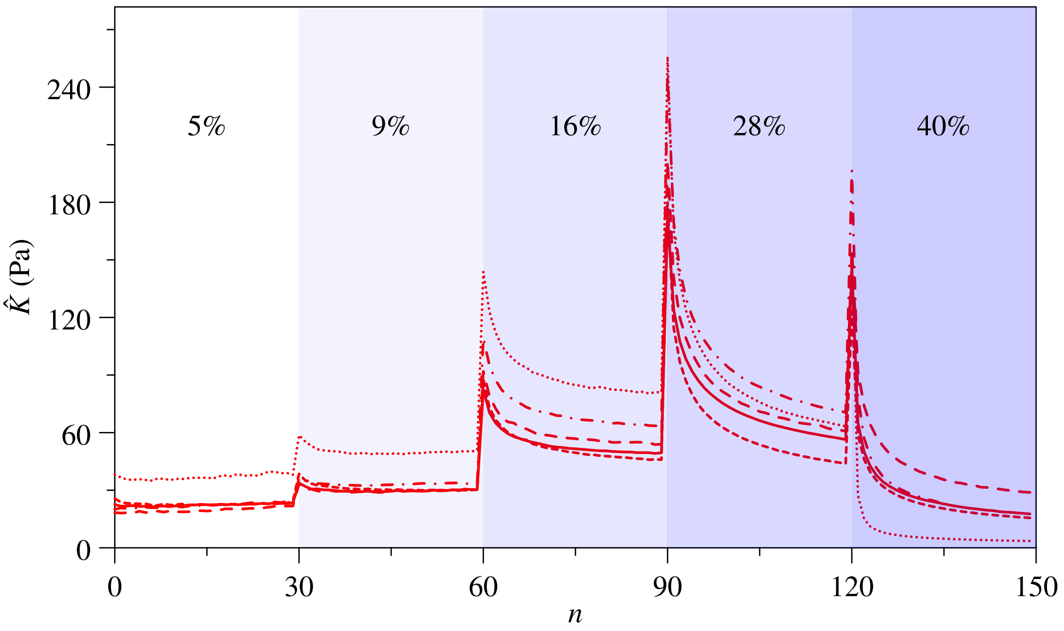

Figure E. Reduced nonlinear modulus $\hat{K}$ (ratio of stress amplitude and strain amplitude attained during one cycle, see also Fig. 2 in the main text) of F-actin/HMM networks. Nonlinear response to the nonlinear oscillations in the main text for five identically prepared samples. All five curves exhibit the features of stationary and dynamic stiffening and softening. For one sample (dotted line), the nonlinear modulus $\hat{K}$ dramatically decreases at 40 % strain amplitude. This may be indicative of the F-actin network detaching from the rheometer plates, which is often observed for F-actin networks at large strains [5]. The sudden breakdown stands out from the much gentler shakedown in all other samples.

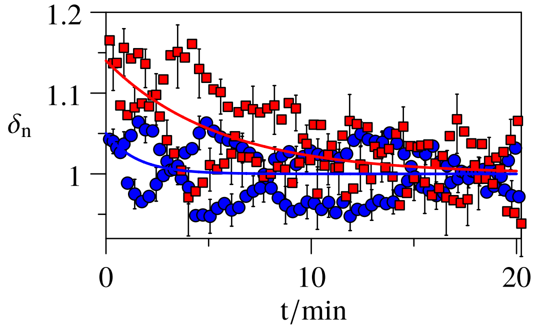

Figure F. Recovery of the normalized loss angle after a transient shear pulse of an amplitude of 10% (circles) and 30% (squares), corresponding to the stiffness data in Fig. 3 in the main text. Solid lines represent exponential fits. Zero time marks cessation of the strain pulse. Normalization according to the procedure described in Materials and Methods.

References

1. Wolff L, Fernandez P, Kroy K (2010) Inelastic mechanics of sticky biopolymer networks. New Journal of Physics 12: 053024.

2. Alberts B, Johnson A, Lewis J, Reff M, Roberts K, et al. (2002) Molecular Biology of the Cell. Garland Science, 4th ed. edition.

3. Trepat X, Deng L, An SS, Navajas D, Tschumperlin DJ, et al. (2007) Universal physical responses to stretch in the living cell. Nature 447: 592–5.

4. Mizrahi N, Zhou E, Lenormand G, Krishnan R, Weihs D, et al. (2012) Low intensity ultrasound perturbs cytoskeleton dynamics. Soft Matter 8: 2438–2443.

5. Schmoller KM, Fernández P, Arevalo RC, Blair DL, Bausch AR (2010) Cyclic hardening in bundled actin networks. Nature Communications 1: 134.
